# Mechanisms of chemotherapy-induced behavioral toxicities

**DOI:** 10.3389/fnins.2015.00131

**Published:** 2015-04-21

**Authors:** Elisabeth G. Vichaya, Gabriel S. Chiu, Karen Krukowski, Tamara E. Lacourt, Annemieke Kavelaars, Robert Dantzer, Cobi J. Heijnen, Adam K. Walker

**Affiliations:** Laboratory of Neuroimmunology, Division of Internal Medicine, Department of Symptom Research, The University of Texas MD Anderson Cancer CenterHouston, TX, USA

**Keywords:** chemotherapy, inflammation, fatigue, neuropathy, cognition, DAMP, cellular metabolism, mitochondria

## Abstract

While chemotherapeutic agents have yielded relative success in the treatment of cancer, patients are often plagued with unwanted and even debilitating side-effects from the treatment which can lead to dose reduction or even cessation of treatment. Common side effects (symptoms) of chemotherapy include (i) cognitive deficiencies such as problems with attention, memory and executive functioning; (ii) fatigue and motivational deficit; and (iii) neuropathy. These symptoms often develop during treatment but can remain even after cessation of chemotherapy, severely impacting long-term quality of life. Little is known about the underlying mechanisms responsible for the development of these behavioral toxicities, however, neuroinflammation is widely considered to be one of the major mechanisms responsible for chemotherapy-induced symptoms. Here, we critically assess what is known in regards to the role of neuroinflammation in chemotherapy-induced symptoms. We also argue that, based on the available evidence, neuroinflammation is unlikely the only mechanism involved in the pathogenesis of chemotherapy-induced behavioral toxicities. We evaluate two other putative candidate mechanisms. To this end we discuss the mediating role of damage-associated molecular patterns (DAMPs) activated in response to chemotherapy-induced cellular damage. We also review the literature with respect to possible alternative mechanisms such as a chemotherapy-induced change in the bioenergetic status of the tissue involving changes in mitochondrial function in relation to chemotherapy-induced behavioral toxicities. Understanding the mechanisms that underlie the emergence of fatigue, neuropathy, and cognitive difficulties is vital to better treatment and long-term survival of cancer patients.

## Introduction

When someone describes his/her battle with cancer, the discussion inevitably intertwines their experience of the disease with their experience of the treatment. This is because the toxicities of cancer treatment are commonly debilitating and can drastically reduce quality of life. Indeed, often these side effects persist for weeks, months, or years after patients are cancer-free. Furthermore, symptoms can be so severe that physicians may be forced to deviate from the optimal treatment strategy for a patient, which can directly influence survival.

It has also been found that high symptom expression is related to increase risk of mortality. For example, Innominato et al. (2013) found fatigue to be a negative predictor of survival of metastatic cancer which highlights the importance of studying symptoms to both improve quality of life of cancer patients and potentially impact survival.

While there are many anti-cancer drugs used with widely varying mechanisms of action, there appear to be a common set of symptoms induced by many of these agents which include fatigue, cognitive dysfunction, and peripheral neuropathy (Cleeland et al., 2003). No FDA-approved treatment is currently available for treatment or prevention of these symptoms. In addition, the underlying mechanisms of chemotherapy-induced symptoms are poorly understood. The current dogma of the mechanisms responsible for the symptoms of chemotherapy largely revolves around neuroinflammation (Cleeland et al., 2003; Miller et al., 2008; Dantzer et al., 2012). This has primarily been driven by preclinical and clinical studies in non-cancer contexts demonstrating that propagation of peripheral inflammatory signals to the brain results in acute behavioral symptoms of sickness which can transition into chronic conditions. For instance, it is clear that there is a temporal dissociation between the symptoms of sickness and the development of persistent cognitive, neuropathic or mood, and behavioral changes after the illness has dissipated (Capuron et al., 2002). During the acute phase response to a disease and/or inflammatory response, reduced mood, increased pain and fatigue are adaptive processes to aid in the recovery from illness. However, when these symptoms remain after the disease has cleared then they have transitioned into a chronic and pathological condition (Walker et al., 2014). Such findings made neuroinflammation an attractive mechanistic target to explain the behavioral toxicities in response to cancer and chemotherapy given that many of the side-effects of chemotherapy remain long after treatment has ceased.

On the basis of the data on inflammation-induced behavioral phenomena, a great deal of research into the symptoms of cancer and chemotherapy has focused on peripheral and central cytokine signaling as a possible common inducer of these toxicities as well. However, cancer and its treatment appear to exist as a particularly unique circumstance. Cancer-related neuroinflammation may be a consequence of peripheral inflammatory signaling due to the effect of therapy on the tumor or other peripheral tissues or may be a direct consequence of chemotherapy agents localizing to cells of the central nervous system (CNS) (Giurgiovich et al., 1997; Cavaletti et al., 2001). Now after over a decade of research on the role of neuroinflammation in chemotherapy-induced symptoms, it is imperative to re-evaluate the available evidence for the role of neuroinflammation in chemotherapy-induced symptoms. Doing so will provide a clear account of what we have learned and an understanding of where we are heading. In this review we will discuss the role of neuroinflammation in chemotherapy-induced fatigue, cognitive dysfunction, and peripheral neuropathy and pain, as well as highlight potential novel mechanistic candidates for future investigation. We recognize that the relationship between chemotherapy-induced symptoms and cancer-related symptoms is complex. Based on the current literature and minimal data for pre-diagnosis and treatment naïve patients the two cannot be fully disentangled. However, much of what is known is derived from studies carried out in non-tumor bearing rodents treated with chemotherapy. Although these symptoms are apparent in patients with both CNS and non-CNS cancers, CNS cancers hamper the study of the specific effects of chemotherapy because of the possible confounding effects of the tumor. To avoid such confusion, we will focus our discussion on the relationship between neuroinflammation and symptoms in non-CNS cancer patients. Similarly, additional symptoms such as cachexia/ anorexia induced both by the cancer and chemotherapy are thought to be regulated by central cytokine signaling (reviewed in Illman et al., 2005) and probably potentiate neuroinflammation and chemotherapy-induced fatigue, cognition and neuropathy. This interplay alone could serve as a topic for review. We have decided therefore, to limit the scope of this review specifically to what is known about chemotherapy-induced fatigue, cognitive dysfunction and neuropathy/pain.

## Neuroinflammation in chemotherapy-induced behavioral toxicities

### Fatigue

Fatigue is one of the most common symptoms experienced by cancer patients (Cleeland, 2007). In some studies up to 60% of patients receiving chemotherapy have been found to exhibit symptoms of fatigue (Bock et al., 2014). While the experience of fatigue often declines shortly after treatment, for many survivors their fatigue persists long after treatment cessation. Indeed, it is estimated that between 19 and 38% of cancer survivors still suffer from fatigue after treatment has stopped (Cella et al., 2001; Prue et al., 2006; Berger et al., 2010). Fatigue significantly impairs one's quality of life by exerting its effects at the physical, psychological, and social levels (Curt, 2000). While the term fatigue has become common parlance, many of us take for granted the complexity of discrete neurological and biobehavioral components that comprise it. At a basic level fatigue can be divided into peripheral fatigue and central fatigue (Davis, 1995; Chaudhuri and Behan, 2000). Peripheral fatigue refers to physical exhaustion and is often described in terms of muscle fatigue and lack of physical energy. Central fatigue refers to the set of discrete central processes that drive the cognitions associated with fatigue, which include a lack of motivation to engage in a given behavior. When studies also assess the motivational components of fatigue, the incidence of fatigue in cancer patients and survivors rises to 50% or higher (Curt et al., 2000; Sadler et al., 2002; Van Belle et al., 2005). Understanding the discrete units of central fatigue is complicated and only recently, the topic has entered the forefront of scientific pursuit. A consideration of fatigue cannot avoid mentioning the high degree of convergence between fatigue and depression. Fatigue is indeed part of the diagnostic criteria for depression, and approximately 73% of patients with depression report a lack of energy and fatigue (Lecrubier, 2006). These rates are even higher in cancer patients experiencing depression, with somatic depression-related symptoms being reported as more prominent than affective symptoms (Wedding et al., 2007). A meta-analysis by Brown and Kroenke (2009) revealed an overall correlation of 0.56 between fatigue and depression in patients with cancer. This indicates that while fatigue and depression are related, they still do have independent components. This is further evidenced by studies indicating that the progression for fatigue and depression are different over the course of treatment in patients (Visser and Smets, 1998; Brown and Kroenke, 2009). At the methodological level, the overwhelming majority of studies that have investigated the link between cancer and its treatment with fatigue rely on patient self-report of fatigue on an unidimensional scale, therefore, omitting any consideration of the various components of fatigue. Below we will describe what has been discovered in regards to fatigue in preclinical and clinical models for cancer and chemotherapy in relation to neuroinflammation.

Neuroinflammation has been overwhelmingly proposed as the mechanism to account for cancer-related fatigue (Dantzer et al., 2014). This has partly been driven by evidence for a role of neuroinflammation in fatigue in patients from non-cancer contexts such as rheumatoid arthritis and multiple sclerosis. However, understanding the mechanisms underlying fatigue in cancer patients receiving chemotherapy may require a completely different mechanism of induction and/or maintenance than inflammation-induced fatigue. For many studies, particularly those at the clinical level, dissociation between chemotherapy-induced fatigue vs. that induced by the disease or by additional treatment strategies is difficult. In contrast, few preclinical studies investigate the synergistic effect of the disease and chemotherapy on fatigue, but choose to most often look at each in isolation. One murine study, however, did examine fatigue-related behaviors in mice with non-inflammatory Lewis Lung Carcinoma cell tumors that received the chemotherapeutic agent Etoposide (Wood et al., 2006). Etoposide significantly reduced voluntary wheel running activity used as an index of fatigue despite its intrinsic complexity (Novak et al., 2012) with a concomitant increase in serum IL-6 but causation cannot be inferred.

Human studies allow us to infer exacerbation of symptoms by chemotherapy on existing fatigue in cancer patients. For example, a recent study showed that children with acute lymphoblastic leukemia had reduced muscle strength, bone density, and fitness at diagnosis prior to treatment (Ness et al., 2014). However, the severity of these symptoms did not appear as great as those that were observed in such patients following treatment with chemotherapy, which is suggestive of a significant role of chemotherapy in the development of these symptoms. It should be noted that children receiving chemotherapy for acute lymphoblastic leukemia also receive high doses of the synthetic glucocorticoid dexamethasone which is likely to also contribute to symptoms of fatigue.

Additionally, most clinical studies that included investigation of chemotherapy-related fatigue relied upon the measurement of peripheral markers of inflammation as a proxy for central inflammatory processes. For example, Wang et al. (2012) found that fatigue as measured by the fatigue item of the MD Anderson Symptom Inventory (MDASI) was positively associated with serum interleukin (IL)-6 and soluble tumor necrosis factor-receptor 1 (sTNF-R1) concentrations for colorectal and oesophageal cancer patients treated with combined chemotherapy and radiotherapy. Importantly, symptoms peaked at the end of treatment suggestive of the cumulative effects of treatment toxicity. More specific to chemotherapy alone, Pertl et al. (2013) investigated fatigue and depression symptoms in patients with breast cancer. The acute phase protein C-reactive protein (CRP) at baseline predicted changes in fatigue as measured by Functional Assessment of Cancer Therapy—Fatigue Scale in patients receiving chemotherapy and was independent of depression. It should be noted that other inflammatory markers including the cytokines interferon (IFN)-γ, IL-6 and TNF-α were assessed and no such relationship with fatigue emerged. However, circulating levels of cytokines are often very low and close to undetectable in many cases making it hard to draw firm conclusions. Nevertheless, the relationship with CRP may indicate the importance of the baseline level of inflammatory activity to predict fatigue severity in response to chemotherapy. A recent murine model was used to investigate the development of fatigue, as measured by decreased voluntary wheel running in response to systemic injection of cyclophosphamide, doxorubicin, and fluorouracil (Weymann et al., 2014). Reduced wheel running and elevated pro-inflammatory cytokine expression in the brain were observed which were attenuated with a central injection of orexin—a neuropeptide responsible for arousal and wakefulness. While the data provide compelling evidence of a role for neuroinflammation in chemotherapy-induced fatigue, it is important to note that these effects are not observed across all studies and that not all chemotherapeutic agents induce inflammation.

Research into fatigue prevalence in survivors has also been conducted, and gives us some insight into the transition from acute symptoms of treatment to long-term fatigue. Researchers recently, followed breast cancer patients from just prior to adjuvant chemotherapy through to 1 year post-treatment (Moore et al., 2014), and noted a tendency for patients to report high levels of fatigue at baseline which worsened during chemotherapy and had not fully resolved by 1 year post-treatment. Such a finding is not uncommon and many studies cite evidence of inflammation as a contributing factor. Alfano et al. (2012) found that breast cancer survivors had a 1.8 fold greater chance of suffering from fatigue if they exhibited high serum CRP levels. Moreover, higher CRP levels showed a significant positive correlation with higher scores for behavioral, sensory, and total fatigue on the Piper Fatigue Scale. Fatigue in breast cancer survivors has also been shown to correlate positively with peripheral CRP levels and leukocyte counts but not with IL1-receptor antagonist (RA), IL-6, and soluble TNF-Receptor1 (sTNF-R1), which again diminishes the role of a cytokine-specific mechanism. Collado-Hidalgo et al. (2006) compared the *ex vivo* monocyte response to lipopolysaccharide (LPS) between breast cancer survivors with chronic fatigue and those without. The *ex vivo* response of peripheral monocytes to LPS was significantly greater for survivors with fatigue compared to their control counterparts.

The question remains, however, what causes the transition from acute symptoms to chronic fatigue after chemotherapy—and where might inflammation fit into this transition? Smith et al. (2014) attempted to answer this question. They hypothesized that inflammation may persist into survivorship and cause chronification of fatigue via changes to the epigenome. They looked at DNA methylation patterns of peripheral blood mononuclear cells in response to chemotherapy in breast cancer patients. They were able to observe an association between plasma sTNFR1 and fatigue but no epigenetic mechanism could be supported by the data. Reinertsen et al. (2011) investigated single nucleotide polymorphisms (SNPs) for IL-1β and IL-6R but found no relationship with fatigue. Hence, while there is compelling evidence to implicate neuroinflammation with fatigue emergence during and after a variety of chemotherapy agents, it has not been possible so far to demonstrate causation. Identifying cause-and-effect relationships between chemotherapy and behavioral toxicities is further complicated by the widely varying mechanisms of action of different chemotherapeutic agents. For instance, inflammation is a likely candidate for etoposide-induced fatigue as it activates p38 MAPK pathway (Wood et al., 2006), while bortezomib inhibits NF-kB (Ma et al., 2003; Mitsiades et al., 2006), and therefore, would not be expected to induce an inflammatory response. Despite the variations in the degree to which different chemotherapeutic agents induce inflammation, fatigue appears to remain a constant and common outcome of chemotherapy. The reason for this may lie in the possibility that treatment-related fatigue is not primarily or solely caused by inflammatory mediators, but is induced by treatment-induced intracellular metabolic changes in the target tissue such as direct mitochondrial damage (discussed below).

### Cognitive dysfunction

Chemotherapy-induced cognitive impairment (CTCI), also referred to as “chemobrain” or “chemofog,” is experienced by 15–80% of cancer patients and survivors (Cleeland et al., 2003). The variance in incidence rates of CTCI can be attributed to different treatment modalities as well as methodological variations across studies such as use of different definitions, objective vs. subjective tests of CTCI, and times of assessment of CTCI (Hutchinson et al., 2012; O'farrell et al., 2013). The most robust effects of chemotherapy are reported for executive function, memory, and processing speed (Cleeland et al., 2003; Jones et al., 2013; Seretny et al., 2014)—all of which involve frontal regions of the brain. Brain imaging studies indeed show subtle reductions in white and gray matter volume and density and frontal hypo—as well as hyperactivity during memory-related cognitive tasks in chemotherapy treated breast cancer survivors (Wieseler-Frank et al., 2005; Hutchinson et al., 2012; O'farrell et al., 2013). While these changes in brain volume and activity improve over time after cessation of treatment, subtle changes are still apparent years into survivorship (Jounai et al., 2012). Several mechanisms underlying cognitive impairment have been proposed including direct neurotoxic injury, decreased neurogenesis, hormonal pathways, and neuroinflammation (Seigers et al., 2013). Neuroinflammation as a possible explanatory mechanism for cognitive dysfunction has been studied both in clinical and animal studies.

Several clinical studies have now been published that focus on the relation between peripheral inflammatory markers, as a proxy for neuroinflammation, and cognitive performance (see Seretny et al., 2014 for a recent review). Overall, results from these studies tentatively point to a role for inflammation in CTCI (Seretny et al., 2014). Ganz et al. (2013) reported an association between soluble TNF receptor type II (sTNF-RII), a marker for TNF-α activity, and subjective memory complaints in breast cancer survivors. Higher levels of sTNF-RII were associated with greater memory complaints approximately 3 months post treatment and a decrease in sTNF-RII over the 12 months post treatment was related with improvements in self-reported memory. Of note, the observed relation between sTNF-RII and subjective complaints disappeared when controlling for fatigue, suggesting an intertwining of self-reported fatigue and cognitive symptoms. Reporting on a subset of the same breast cancer survivor sample, Pomykala et al. (2013) showed a positive association between several cytokine markers (among which sTNF-RII) and subjective memory complaints as well as cerebral metabolism both at 3 and 12 months post treatment. Janelsins et al. (2012), reporting on a different cohort, found an association between increases in the chemokine MCP-1 during two cycles of doxorubicin-based chemotherapy and less subjective cognitive problems at the end of the two cycles in breast cancer patients. Although not significant, increases in the cytokines IL-6 and IL-8 were associated with more subjective cognitive problems, suggesting that the relation between CTCI and inflammation is intricate and might not readily be captured with the assessment of single inflammatory markers. In the same study, no association was found between any of the inflammatory markers and subjective cognitive difficulties in breast cancer patients treated with a methotrexate-based chemotherapy cocktail, indicating that the relation between inflammation and CTCI might be chemotherapy-agent-specific. Kesler et al. (2013) reported an interaction between IL-6 and TNF-α on performance on a verbal learning test in chemotherapy-treated breast cancer survivors. IL-6 and TNF-α were also related to lower left hippocampal volume, suggesting that inflammation possibly reduced cognitive function through effects on the hippocampus. On the other hand, Gan et al. (2011) did not observe any relationship between objectively assessed cognitive function and inflammatory biomarkers in head and neck cancer survivors. However, considering the small sample size of this study (*n* = 10), this null finding needs to be interpreted with caution.

The above described peripheral markers of inflammation are considered a proxy for neuroinflammation and indeed seem to be associated with brain metabolism and volume, implicating that the peripheral markers are representative of a central mechanism. However, the use of more direct measures of neuroinflammation, such as inflammatory markers in cerebrospinal fluid or assessment of microglia activation with positron emission tomography (Dickens et al., 2014) would significantly increase our understanding of the role of neuroinflammation in CTCI. Of course, such measures are not always feasible due to their invasiveness for the patient and high costs. Clinical studies also do not allow for an easy disentanglement of the effects of the tumor and its treatment on subsequent cognitive difficulties. There is evidence of disease-driven cognitive dysfunction, such that subtle cognitive impairments accompanied by subtle differences in brain volume and activity are already apparent before the start of chemotherapy (Cleeland et al., 2003; O'farrell et al., 2013). Furthermore, an association between inflammatory markers and cognitive impairment has also been observed prior to chemotherapy (Bernard et al., 2012). These disease-driven impairments and their possible association with inflammation can be addressed with longitudinal study designs that incorporate assessments prior to the onset of chemotherapy. Such studies have already been undertaken with regard to CTCI showing the feasibility of these designs but, unfortunately, measures of inflammation have not yet been included.

Animal studies do allow for the study of the effects of chemotherapy alone (i.e., without tumor interference) and also for more direct measures of neuroinflammation through the assessment of cytokines concentrations in the brain and microglia activation. Another advantage of the use of animal models is the relatively easy assessment of both the acute and long-term neuroinflammatory response to chemotherapy. In most rodent studies published up to now, measures of inflammation served as a secondary outcome and more direct, mechanistic investigations between neuroinflammation and chemotherapy-induced cognitive dysfunction are required. Nevertheless, results from rodent studies do suggest that neuroinflammation might be related to cognitive dysfunction in specific chemotherapy models (Lecrubier, 2006).

Seigers et al. (2010) reported an increase in the number of active microglia in the hippocampus 1 and 3 weeks after methotrexate treatment. However, they did not find an effect of methotrexate on cytokine levels in the hippocampus or on microglial activation as assessed by PET ([11C]PK11195). Furthermore, methotrexate appeared to reduce peripheral levels of cytokines (Topp et al., 2000). The latter finding is not surprising considering the anti-inflammatory properties of methotrexate (Cutolo et al., 2001) and indicates that the observed increase in the number of active microglia may represent activation of anti-inflammatory M2 microglia (Cherry et al., 2014). Briones and Woods (2014) showed that treatment with a combination of cyclophosphamide, methotrexate, and fluorouracil led to an increase in IL1-β and TNF-α in the corpus callosum of rats and a decrease in the anti-inflammatory cytokine IL-10 approximately 4 weeks after chemotherapy. These changes in cytokine levels were accompanied by reduced performance on a working memory task. Administration of a COX-2 inhibitor normalized the cytokine concentrations and attenuated the deficit seen in cognitive performance, strengthening the assumption of a direct relationship between the observed neuroinflammation and cognitive impairment. Findings from this animal study stand in contrast to Janelsins' report on patients receiving the same combination of chemotherapeutic agents in whom no increase in peripheral markers of inflammation were observed (Janelsins et al., 2012), possibly indicating that the neuroinflammation found in animals cannot be translated to peripheral inflammation. Impaired performance in a working/spatial memory task was also observed in rats treated with either cyclophosphamide or doxorubicin 3 weeks prior to assessment of cognitive performance. Cyclophosphamide only led to inflammation in the hippocampus assessed as an increased number of activated microglial cells (Dina et al., 2001). Finally, microglial activation throughout the brain was observed in one out of ten mice treated with fluorouracil (Schaefer, 2014) at 1 day post-treatment. Cognitive performance was not assessed in this study.

In sum, clinical studies indicate that peripheral inflammation might be related to cognitive impairments after chemotherapy, suggesting a role for neuroinflammation in CTCI. This notion is corroborated by findings from animal models showing that chemotherapy can lead to both neuroinflammation and impairments in cognitive function. Interestingly, these associations are observed immediately as well as some weeks after therapy. Both clinical and animal studies indicate that a neuroinflammatory mechanism underlying CTCI is probably restricted to specific chemotherapeutic agents, stressing the importance of studying CTCI in different patient populations and models of chemotherapy.

### Neuropathy

Peripheral neuropathy characterized by pain, numbness, and temperature sensitivity is another common side effect of chemotherapy known as chemotherapy-induced peripheral neuropathy (CIPN) (Dougherty et al., 2004; Wolf et al., 2008). CIPN occurs in about 60% of cancer patients (Rowinsky et al., 1993a,b; Windebank and Grisold, 2008; Wolf et al., 2008; Cavaletti et al., 2011; Seretny et al., 2014) and can cause dose limitations or early cessation of treatment making it a challenge for effective cancer treatment (Cavaletti et al., 1992; Uhm and Yung, 1999; Polomano and Bennett, 2001; Mielke et al., 2006). As reported for fatigue and cognitive deficits, CIPN can persist after completion of treatment thereby contributing to the reduction in quality of life of cancer survivors.

Chemotherapy-treated individuals frequently report an acute pain phase in the days immediately following treatment (Gamelin et al., 2002; Grothey et al., 2011; Park et al., 2011). This acute phase usually subsides. However in some cases acute CIPN symptoms transition into a chronic pain phenotype (Seretny et al., 2014). Both the acute and chronic CIPN symptoms can be problematic for patients. Intense acute pain symptoms can lead to the necessity of decreasing the dose of the drug or number of treatment cycles. Persistent chronic pain states can also adversely affect quality of life both during and following completion of chemotherapy treatment (Vichaya et al., 2013). CIPN symptoms are most frequently reported in a “glove and stocking” distribution in which patients report neuropathy symptoms in their hands and feet (Kim et al., 2015). These neuropathy symptom profiles are reported across different classes of chemotherapeutic agents including taxanes, platinum, proteasome inhibitors, and vinca-alkaloids. Why many different chemotherapeutic agents result in similar neuropathy profiles is unclear. More importantly molecular/cellular cause(s) of CIPN remain unknown.

In this section we shall highlight research on inflammation as a potential cause of CIPN. Human studies discussed above measured chemotherapy-induced increases in peripheral pro-inflammatory cytokine levels corresponding with behavioral toxicities such as cognitive deficits, fatigue, and neuropathy. Animal studies have enabled investigators to further elucidate effects of inflammation on neuronal tissues such as peripheral sensory neurons as a potential cause of CIPN. Several investigators have measured increased pro-inflammatory cytokines, such as IL-1β, IL-6, and TNF-α, at the site of peripheral sensory neurons (either in the dorsal root ganglia or spinal cord) of chemotherapy treated rodents (White et al., 2005; Ledeboer et al., 2007; Xiao et al., 2011; Wang et al., 2012; Zhang et al., 2012, 2013; Pevida et al., 2013; Janes et al., 2014a). Studies in inflammatory pain have shown that endogenous or exogenous increases in pro-inflammatory cytokines can sensitize peripheral sensory neurons leading to spontaneous discharge and neuropathic pain in the absence of chemotherapy treatment (Topp et al., 2000; Dina et al., 2001; Wieseler-Frank et al., 2005; Schafers and Sorkin, 2008). Due to the negative effects that pro-inflammatory cytokines have on peripheral sensory neurons, cytokines were investigated in the context of CIPN. It quickly became clear that pro-inflammatory cytokines were actively contributing to chemotherapy-induced neuropathic symptoms as blockade via cytokine antagonists such as IL-1 receptor antagonist or anti-TNF-α attenuated chemotherapy-induced neuropathy (Ledeboer et al., 2007; Cata et al., 2008; Ale et al., 2014). Furthermore, these pro-inflammatory cytokine effects could be regulated through changing the pro-inflammatory vs. anti-inflammatory cytokine balance at neuronal tissue sites. Ledeboer et al. (2007) demonstrated that intrathecal administration of the anti-inflammatory cytokine IL-10 could attenuate paclitaxel-induced neuropathy. Another group also found that increasing anti-inflammatory cytokine levels, IL-10 and IL-4, in the spinal dorsal horn via an S1PR_1_ antagonist could also prevent CIPN in rodents (Janes et al., 2014a). Others have shown that thalidomide, a biological agent shown to inhibit TNF-α, reduced chemotherapy and bone cancer induced neuropathy (Cata et al., 2008; Gu et al., 2010) in rodent models. Conversely, when thalidomide was used in the treatment of multiple myeloma in patients, thalidomide administration induced neuropathic symptoms (Mileshkin et al., 2006; Chowdhury et al., 2013). For the most part studies have demonstrated that increases in pro-inflammatory cytokines either in the dorsal root ganglia or spinal cord corresponds with symptoms of CIPN. Prevention of these pro-inflammatory cytokines can attenuate neuropathy symptoms. However, the therapeutic effect of inhibition of these cytokines in humans has yet to be attained.

These initial discoveries were highly supportive of the hypothesis that CIPN can be driven by an inflammatory mechanism and drove researchers to investigate which specific cell type(s) were responsible for chemotherapy-induced production of pro-inflammatory cytokines. Monocytes/macrophages, a component of the innate immune system, are major producers of peripheral pro-inflammatory cytokines during infection and at injury sites. Neuronal cells have also been shown to produce pro-inflammatory cytokines as well as chemokines. Zhang et al. (2013) found that chemotherapy induced the production of monocyte-chemoattractant-protein-1 (MCP-1, also known as CCL2) in murine DRGs, which corresponded with macrophage infiltration of the DRGs. It was also shown that blockade of MCP-1 prevented macrophage infiltration and symptoms of CIPN (Pevida et al., 2013; Zhang et al., 2013). Furthermore, treatment with minocycline, an FDA-approved antibiotic also known to inhibit macrophages as well as pro-inflammatory cytokine production, prevented CIPN across a range of chemotherapeutic agents in murine systems (Boyette-Davis and Dougherty, 2011; Boyette-Davis et al., 2011; Drouin-Ouellet et al., 2011; Gwak et al., 2012). The pre-clinical positive results on the use of minocycline in CIPN prevention has led to current clinical trials investigating the efficacy of minocycline in the prevention of CIPN in patients. The success of macrophage/microglia blocking agents in prevention of CIPN was unexpected as chemotherapy administration has mainly been shown to induce astrocyte activation but not microglia activation in DRGs and spinal cord (Di Cesare Mannelli et al., 2013, 2014; Janes et al., 2014b; Robinson et al., 2014).

Chemotherapy administration has been shown to greatly reduce the density of intraepidermal nerve fibers (IENFs) crossing the basement membrane into the epidermis (Dougherty et al., 2004; Boyette-Davis and Dougherty, 2011; Boyette-Davis et al., 2011; Kosturakis et al., 2014; Mao-Ying et al., 2014). This reduction, but not total loss of IENFs is hypothesized to leave remaining neurons highly sensitized and a potential reason for neuropathic outcomes. It is unclear what leads to IENF retraction. Some researchers propose it to be the result of altered mitochondrial function and energy states in the sensory neurons (discussed below). Others have suggested that the nerve terminals are the most vulnerable part of sensory neurons and therefore, most easily damaged by chemotherapy administration (Miltenburg and Boogerd, 2014). Chemotherapy-induced increases in cytokine levels or macrophage infiltration at nerve terminals has yet to be investigated.

## Alternative mechanisms for chemotherapy-induced behavioral toxicities

Above we have presented evidence in support of the role of chemotherapy-induced neuroinflammation in the symptoms of fatigue, cognitive dysfunction, and neuropathy. There is certainly evidence to indicate that neuroinflammation is involved in each of these symptoms. However, there is limited evidence to support a causal relation between neuroinflammation and these chemotherapy-induced symptoms, calling for the consideration of additional pathways.

### Damage-associated molecular patterns

Damage-associated molecular patterns—also known as danger-associated molecular patterns, cell death-associated molecules, or DAMPs—are endogenous intracellular molecules released due to compromised membrane integrity during cellular death and injury (Kaczmarek et al., 2013). DAMPs can activate membrane receptors like the receptor for advance glycation end product (RAGE) and pattern recognition receptors (PRRs), such as toll-like receptors (TLRs), NOD-like receptors (NLRs), and purinergic receptors on target cells to initiate inflammatory responses (Chen and Nunez, 2010). Coincidently, TLRs and NLRs also recognize pathogens and are a shared pathway for infectious and non-infectious inflammation (Pradere et al., 2014). Most often released as the result of decreased plasma membrane integrity of injured cells, DAMPs can be classified as proteins (Rubartelli and Lotze, 2007), nucleic acids (Bernard et al., 2012; Jounai et al., 2012; Paludan and Bowie, 2013), purines (Schaefer, 2014), and other non-protein molecules such as reactive oxygen species (ROS). Interestingly, many of the DAMPS that are released during necrosis as well as their receptors are also overexpressed in tumor cells (Castellani et al., 2014). Here, we examine function of the high-mobility group box-1 (HMGB1) protein, DNA and RNA fragments, purines such as adenosine triphosphate (ATP) and adenosine, and ROS and provide possible links to tumorigenesis and chemotherapeutic agents.

#### High-mobility group box-1

HMGB1 is perhaps the best characterized DAMP. Synthesized as a nuclear protein, HMGB1 is normally bound to DNA acting as a transcription factor and is released during cellular damage or injury. It is released less during programmed cell death or apoptosis where the up-regulation of histone 2B inhibits the dissociation of HMGB1 from DNA (Lotze et al., 2007). Extracellular HMGB1 can promote angiogenesis, stem cell migration, as well as neutrophil recruitment and subsequent pro-inflammatory immune responses via the activation of TLR2, TLR4, and RAGE. Conversely, activated T-cells or natural killer cells (Lotze and Tracey, 2005) as well as many chemotherapeutic agents promote the release of HMGB1 from tumor cells and healthy tissues (Tang et al., 2010). Hence HMGB1 liberation may be promoted by chemotherapy-induced cell death. Additionally, HMGB1 can also activate numerous immune cells including macrophages and dendritic cells via TLR and RAGE to stimulate the release of cytokines such as TNF-α, interleukin (IL)-1α, IL-1β, and IL-6 (Lotze and Tracey, 2005). Therefore, HMGB1 likely contributes to the elevations in inflammatory markers observed in patients treated with chemotherapy.

HMGB1 has been linked to muscle function and strength and, therefore, could play a role in peripheral fatigue (Grundtman et al., 2010). Furthermore, several studies have indicated a role for HMGB1 release in the development of non-chemotherapy-induced neuropathies, such as nerve injury (Shibasaki et al., 2010; Feldman et al., 2012) and cognitive impairment following surgery or sepsis (Chavan et al., 2012; Li et al., 2013; Vacas et al., 2014). While HMGB1 has not yet directly been shown to mediate these symptoms in the context of chemotherapy, the known release of HMGB1 in response to many chemotherapeutic agents indicates that research down this avenue is warranted.

#### Reactive oxygen species

Primarily generated in the mitochondria, ROS are produced as a part of normal respiration and energy metabolism. In the physiological state, ROS are rapidly converted to hydrogen peroxide and ultimately to water and oxygen in the cytosolic space which is rich in oxidoreductases and non-protein thiols, such as thioredoxin and glutathione. The accumulation of ROS in the cytosol signals the activation of caspases, mainly caspase-1, via the NLRP3 inflammasome, and subsequently promotes inflammation. Additionally, ROS can also activate the executioner molecule of apoptosis, caspase-3, via the release of cytochrome c and caspase-9 leading to apoptosis (Circu and Aw, 2010).

The intracellular space promotes a reducing environment in healthy cells. During pathological states, the reducing capacity of the cytosol can drastically decrease and thus promote oxidation of many proteins, including HMGB1, and indirectly stimulate the production of secondary DAMP signaling (Lotze et al., 2007). Interestingly, approximately 40% of all FDA-approved anticancer drugs have been shown to induce ROS (Chen et al., 2007). Oxidative stress can produce behavioral toxicities, such as chronic fatigue syndrome (Logan and Wong, 2001; Kennedy et al., 2005), mild cognitive impairment (Fukui et al., 2002; Pratico et al., 2002), and diabetic neuropathy (Nagamatsu et al., 1995; Low et al., 1997; Vincent et al., 2004). Furthermore, there is evidence to suggest that chemotherapy-induced neuropathy (Areti et al., 2014) and cognitive impairment (Aluise et al., 2010) may also be mediated by oxidative stress.

#### Nucleic acids

Classically associated with bacterial or viral infections, nucleic acids such as DNA and RNA can elicit an innate immune response via TLR activation (mainly TLR-3 for double-stranded RNA (Alexopoulou et al., 2001), TLR-7 and 8 for single-stranded RNA (Heil et al., 2004), and TLR-9 for unmethylated DNA (Hemmi et al., 2000). Typically sequestered within the cell, host DNA and RNA are normally considered as unrecognizable by these membrane bound receptors. However, nucleic acids can be released from host cell due to damage or death and can signal as DAMPs. During normal apoptosis nucleotides liberated from membrane-bound organelles are rapidly degraded by nucleases such as DNase and RNase, but during damage or un-programmed cell death, nucleic acids can also be released into the extracellular space as immune stimulators. Furthermore, resident macrophages and dendritic cells can engulf circulating nucleotides to form endosomes (Yasuda et al., 2005) and subsequently stimulate innate immune responses (see review by Ishii and Akira, 2005). Interestingly, mitochondrial DNA (mtDNA) and bacterial DNA are both rich in CpG motifs which is the primary ligand of TLR-9, suggesting that mitochondrial damage induced release of mtDNA can be a potent stimulator of the immune system via TLR-9 activation (Zhang et al., 2010). Platinum-based chemotherapeutic agents, such as cisplatin, target the purine bases of DNA to inhibit replication, transcription, and repair (Jamieson and Lippard, 1999). This may be devastating for the healthy cells of the peripheral and CNS needed to regulate cognition, pain sensation, and behavior. While most neurons are in a post-mitotic state, other cells in the CNS, such as glial cells, still proliferate and are thus susceptible to chemotherapy-induced shortening of telomeres. Therefore, it is conceivable that chemotherapy may accelerate cellular aging leading to senescence and apoptosis (Flanary and Streit, 2004). Furthermore, when cisplatin crosslinks DNA it promotes the cleavage to short nucleic acid fragments and the breakdown of the cell membrane (Barry et al., 1990). Short DNA fragments can leak into the circulation and can act as immunostimulatory agents (Zhang et al., 2010).

Interestingly, the DNA fragmentation that occurs following chemotherapy treatment is also observed in other states of cognitive impairment such as Alzheimer's disease (Lassmann et al., 1995; Stadelmann et al., 1998), aging-related early dementia (Troncoso et al., 1996), and traumatic brain injury (Mattson, 2000). There is a parallel increase in microglia activation and subsequent pro-inflammatory responses (Gehrmann and Banati, 1995). Taken together, these studies indicate that chemotherapy-induced cognitive deficits may be due, in part, to directly increasing DNA damage of neuronal cells, or by promoting accelerated aging via the shortening of telomere.

#### Purines

Purine nucleosides, mainly adenosine and ATP, are physiologically sequestered in the intracellular space and are involved in a multitude of biological functions including energy balance (Leist et al., 1997) and synthesis of nucleic acids (Hartman and Buchanan, 1959). However, extracellular purines are also immunomodulatory and can act as danger signals (Inoue, 2002). Many chemotherapeutic agents elicit anti-tumor effects by stimulating ATP release from tumor cells (Martins et al., 2009), subsequently recruiting dendritic cells (Aymeric et al., 2010) and lymphocytes via P2X7 (an ATP purinergic receptor), and promote phagocytosis and autophagy (Michaud et al., 2011). Furthermore, ATP can also attract monocytes and microglia while simultaneously promoting the production of inflammatory cytokines including IL-1β (Aymeric et al., 2010). Interestingly, increased extracellular ATP concentration has been associated with pain sensation (Tominaga et al., 2001) by the depolarization of sensory neurons (Cook et al., 1997) via the P2X receptors (Rassendren and Ulmann, 2014). Taken together these data indicate that increased extracellular ATP might play a role in CIPN.

Degradation of ATP yields adenosine. Extracellular adenosine concentration drastically increases in response to increased extracellular ATP (Dunwiddie et al., 1997). In many physiological states, adenosine serves as a counter-modulator of synaptic firing by hyperpolarizing neurons (Dulla and Masino, 2013) inhibiting neurotransmitter release (Boison, 2007, 2008) and thus decreasing cerebral activity (Dulla and Masino, 2013). Adenosine also functions as a regulator of sleep and wakefulness in a way that the extracellular concentration of adenosine increases during the waking hours (Huston et al., 1996; Porkka-Heiskanen et al., 1997). Taken together an increase in extracellular adenosine may be an important mediator of chemotherapy-induced fatigue associated with sleep disorders. Indeed, central inhibition of adenosine signaling, via caffeine administration, has been shown to decrease muscle fatigue as well as to increase motor activity (Davis et al., 2003). Furthermore, cognitive disorders such as Alzheimer's (Angulo et al., 2003) and Parkinson's (Schwarzschild et al., 2006) disease are associated with elevated circulating adenosine levels. However, inhibition of adenosine signaling has been associated with cognitive deficits in models of hypoxia (Chiu et al., 2012) and Alzheimer's disease (Dall'igna et al., 2007), as well as with depressive- (Sarges et al., 1990) and anxiety-like behaviors in rodents (Florio et al., 1998; Chiu and Freund, 2014; Chiu et al., 2014).

Finally, it is important to note that extracellular purine is ultimately degraded to uric acid (Becker, 1993). Accumulation and precipitation of uric acid can form monosodium urate crystals to stimulate NOD-like receptors in immune cells and subsequently produce inflammatory cytokines including IL-1β and IL-18 (Gasse et al., 2009). The most obvious example of uric acid-mediated inflammation is gout, where monosodium urate crystals induce arthritis that is characterized by localized pain and inflammation (Martinon et al., 2006; Schumacher et al., 2009). Interestingly, a high plasma uric acid level is also seen after chemotherapy (Liu et al., 2005) and can lead to a high uric acid buildup in both the tumor microenvironment (Hu et al., 2004) and circulation (Liu et al., 2005). Taken together it appears that elevated plasma uric acid after chemotherapy treatment can promote a pro-inflammatory response leading to inflammatory pain. Indeed, studies have shown that acute gout and associated arthritis and inflammatory pain can develop in patients receiving chemotherapeutics such as gemcitabine (Bottiglieri et al., 2013), paclitaxel (Alexandrescu et al., 2009), and capecitabine (Peixoto et al., 2014).

### Cellular metabolism

Chemotherapy is also capable of inducing symptoms by altering the brain's and peripheral nervous system's bioenergetic status. Mitochondria are at the center of cellular bioenergetics as they mediate the production and distribution of ATP. Typically energy production begins with the process of glycolysis within the cytoplasm of a cell. During glycolysis, glucose is broken down into pyruvate. The pyruvate molecules can then either enter the mitochondrial matrix or be converted to lactate. Within the mitochondria, pyruvate is oxidized into citric acid and enters the tricarboxylic acid (TCA) cycle and electron transport chain. Historically it has been thought that lactate formation only occurs in response to a lack of oxygen (i.e., anaerobic conditions) or when there is a disruption in oxidative metabolism. However, despite glucose being considered the primary fuel for normal brain activity (see review by Dienel, 2012), recent evidence suggests that brain lactate production may serve as a signaling molecule and an alternative source of fuel (Gibbs and Hertz, 2008; Suzuki et al., 2011; Tang et al., 2014). Furthermore, lactate produced by the tumor microenvironment serves an important fuel for tumor cell energy metabolism, which is at the basis of the well-known Warburg effect (Pavlides et al., 2009). The interaction between tumor-associated lactate production and brain lactate is still unknown.

#### Association between mitochondrial dysfunction and behavioral changes

The brain is the most energetically demanding organ in the body. Therefore, agents that result in even minor changes in mitochondrial energy metabolism are capable of impacting brain function and producing behavioral changes. For example, there is significant evidence to suggest that mood and psychiatric disorders, such as bipolar disorder, autism, and schizophrenia, are associated with impaired brain energy metabolism (Prabakaran et al., 2004; Young, 2007; Quiroz et al., 2008; Rezin et al., 2009; Rossignol and Frye, 2012). Furthermore, mitochondrial dysfunction has been implicated in the pathophysiology of chronic fatigue syndrome (Myhill et al., 2009, 2013; Murrough et al., 2010) as well as fatigue in patients with multiple sclerosis (Roelcke et al., 1997), and fatigue in rodents treated with an inflammatory agent (Sheng et al., 2011) or exposed to stressors (Tanaka and Watanabe, 2008). For example, higher ventricular lactate levels (an indirect indication of mitochondrial dysfunction) have been observed in patients with chronic fatigue syndrome compared to healthy volunteers (Murrough et al., 2010). Mitochondrial impairment or damage has also been implicated in cognitive impairment such as that associated with aging (Liu et al., 2002; Wang et al., 2006; Liu, 2008), traumatic brain injury (Sauerbeck et al., 2011), HIV-associated dementia (Valcour and Shiramizu, 2004), and Alzheimer's disease (Corona et al., 2010; Dragicevic et al., 2010). HIV/AIDS-related neuropathy (Dalakas et al., 2001) and diabetic peripheral neuropathy (Srinivasan et al., 2000; Chowdhury et al., 2013) have also been associated with mitochondrial damage. Further, there is evidence to suggest that protecting mitochondrial integrity is able to protect against ischemic brain damage as well as the resulting cognitive and motor impairment (Nijboer et al., 2011, 2013).

There is growing evidence that chemotherapy-associated behavioral toxicities are also associated with mitochondrial dysfunction. For example, cisplatin is capable of significantly inhibiting electron chain transport complexes I–IV resulting in a 70% reduction in ATP production (Kruidering et al., 1997). Furthermore, animal models of CIPN show mitochondrial dysfunction within the peripheral nerves and the dorsal root ganglion, axonal mitotoxicity (swollen, vacuolated mitochondria), and poorer antioxidant defense in response to a wide array of chemotherapy agents, including taxanes, vinca alkaloids, platinum agents, and bortezomib (Jin et al., 2008; Melli et al., 2008; Podratz et al., 2011; Xiao et al., 2011; Zheng et al., 2011, 2012). Given that peripheral nerves do not have the protection of the blood brain barrier, it is not unexpected that evidence for mitochondrial dysfunction was first noted here. However, brain mitochondrial function is also affected by chemotherapy. For example, a recent study in patients showed that chemotherapy can induce transient changes in glucose metabolism within the brain (Baudino et al., 2012). Peripheral cisplatin administration was shown to enhance mitochondrial lipid peroxidation levels and protein carbonyl content within the brain of rats (Waseem and Parvez, 2013). Moreover, in a mouse model it has been shown that doxorubicin administration results in an acute reduction in brain complex I function and an increase in pro-apoptotic proteins such as p53 and Bax in brain mitochondria (Tangpong et al., 2006). Finally, it has been shown that doxorubicin treatment increases the susceptibility of rat brain mitochondria to damage from excessive calcium and oxidative stress (Cardoso et al., 2008).

It is important to note that the mitochondrial effects of chemotherapy are often observed in the presence of a tumor. Tumor cells are metabolically demanding and, therefore, have altered metabolic profiles. Furthermore, they can induce metabolic changes that extend to the tumor microenvironment to provide for their metabolic needs (Pavlides et al., 2009; Bonuccelli et al., 2010). Therefore, it is important for future studies to explore how chemotherapy agents affect energy metabolism in the presence of a tumor.

#### Potential mechanisms of chemotherapy-induced mitochondrial dysfunction

While there is growing evidence that chemotherapy is capable of altering mitochondrial function, the mechanism by which this occurs is still unclear. The effect could be an indirect result of increased inflammation and/or oxidative stress or a direct effect of chemotherapy on mitochondria. These potential mechanisms are briefly discussed below.

##### Mitochondria and inflammation

There is both *in vitro* and *in vivo* evidence that mitochondria are sensitive to inflammation. This has been most directly shown by treating cells or mice with the cytokine stimulant, LPS. In both cases significant evidence of mitochondrial metabolic changes were observed (Xie et al., 2004; Hunter et al., 2007). Moreover, decreased brain oxidative phosphorylation has also been observed in a mouse model of sepsis (D'avila et al., 2008). These models induce high levels of inflammation, severe mitochondrial dysfunction, and cellular death (Welty-Wolf et al., 1996; Crouser et al., 2002; Hunter et al., 2007). While this situation is particularly relevant to the symptoms associated with the neurodegeneration observed in Parkinson's disease, the inflammation induced by chemotherapy treatment would likely be markedly milder. Therefore, further investigation is needed to determine if a similar phenomenon is observed in the brain.

##### Mitochondria and oxidative stress

Oxidative stress is an inherent aspect of mitochondrial function. At baseline levels, approximately 1–5% of oxygen used by the cells is converted to ROS (Chance et al., 1979). However, when there is insult to the mitochondria these levels dramatically increase. As mentioned previously, a high proportion of chemotherapeutic agents result in production of ROS. This imbalance in ROS production can lead to cellular damage and mitochondrial damage in particular (reviewed by Adam-Vizi and Chinopoulos, 2006; Areti et al., 2014). Mitochondrial complex I and II of the electron transport chain and mitochondrial DNA (Wallace, 2005) are particularly vulnerable. In addition to expressing genes encoded by the nuclear genome, mitochondria have their own functional genome (mtDNA). The mtDNA has a higher mutation rate than nuclear DNA and a more limited repair capacity than nuclear DNA (Tuppen et al., 2010). This mechanism likely contributes to chemotherapy-induced mitochondrial dysfunction. However, blocking ROS has been shown to be insufficient to prevent cisplatin-induced mitochondrial dysfunction within the kidney (Kruidering et al., 1997) suggesting that chemotherapy may be capable of inducing mitochondrial damage via multiple pathways.

##### Mitochondrial p53

In response to cellular stress there is a rapid accumulation of p53 to the mitochondrial membrane which increases mitochondrial membrane potential, cytochrome c release, and caspase-3 activation (Marchenko et al., 2000). The phosphorylation of p53 by c-Jun N-terminal kinase (JNK) protects p53 from ubiquitination and degradation, thereby enhancing its activity (Fuchs et al., 1998). Using a model of ischemic brain damage, it has been demonstrated that interfering with the mitochondrial JNK/p53 pathway, by inhibiting p53 accumulation [such as with the small molecule inhibitor pifitrin-μ (PFT-μ); (Nijboer et al., 2011)] or by inhibiting the activity of JNK (with the use of TAT-JBD, D-JNKi, and Sab_kim1_; Nijboer et al., 2010, 2013), is neuroprotective and can attenuate damage-associated behavioral deficits. Given that the activity of p53 is a critically involved in chemotherapy-induced tumor cell apoptosis for a wide variety of agents (Pritchard et al., 1997; Hwang et al., 2001; Tan et al., 2002; Bragado et al., 2007), it follows that it is a candidate therapeutic target for the neurotoxic effects of these agents. Further, we have preliminary evidence that that PFT-μ can also inhibit chemotherapy-induced neuropathy (Krukowski et al., 2014, under review).

##### Mitochondrial DNA adducts

Another possible mechanism by which chemotherapy may disrupt mitochondrial function is through the formation of DNA adducts. For example, platinum-based antineoplastic agents act by crosslinking DNA and, consequently, interfering with cellular division and repair, which causes mitochondria to release apoptotic proteins. This effect does not require the formation of adducts between nuclear DNA and cisplatin, but can occur as a direct effect of cisplatin on mtDNA (Yang et al., 2006). Not only can cisplatin-mtDNA adducts form in cancer cells, but these adducts have been noted to develop in other cells throughout the body including the brain (Johnsson et al., 1995; Giurgiovich et al., 1996, 1997). Furthermore, cisplatin has also been noted to accumulate in high levels within the dorsal root ganglion (Mcdonald et al., 2005). This data along with the p53 data would suggest that chemotherapy can induce mitochondrial damage and, consequently behavioral toxicities, via non-inflammation based mechanisms.

### Conclusion

In this review we have evaluated the available evidence for the role of neuroinflammation in chemotherapy-induced behavioral toxicities. Despite neuroinflammation being the clear “mechanism of choice” for many researchers, close examination of the literature forces one to be open to the possibility that other mechanisms also play a critical role, either in conjunction with neuroinflammation or independently. As we point out, clinical studies are rarely designed to allow delineation between inflammatory markers that arise from the cancer vs. those that emerge and dissipate with the start and finish of chemotherapy regimens. This makes it difficult to understand what chemotherapy is precisely doing to the body and brain outside of their effects on tumor progression. On the other hand many preclinical models in the field fail to focus on the causal role of neuroinflammation in many of the symptoms of chemotherapy which leaves us with having to interpret the meaning of associations between central and peripheral markers of inflammation with chemotherapy-induced behaviors. Nevertheless, remarkable progress has been made in the field which places us in an opportune position to assess what we have learned and where we should aim toward.

It is clear that the evidence for neuroinflammation contributing to some symptoms and for particular agents is more convincing than for others. Much more work has been conducted in the field of chemotherapy-induced neuropathy and there is a strong foundation of support for peripheral inflammation as a mediator of pain sensation. More still needs to be done on the central components of pain assessment and experience and inflammation, and many other mechanisms have also been put forward in lieu of neuroinflammation. Much less work has been conducted in the fields of fatigue and cognitive dysfunction following chemotherapy but there remains evidence in favor of the neuroinflammation hypothesis. Unfortunately many studies looking at inflammation and chemotherapy-induced fatigue and cognitive decline report mixed findings and even negative results suggesting that alternative mechanisms need to be considered while also investigating the role of neuroinflammation with greater rigor.

**Figure 1:**
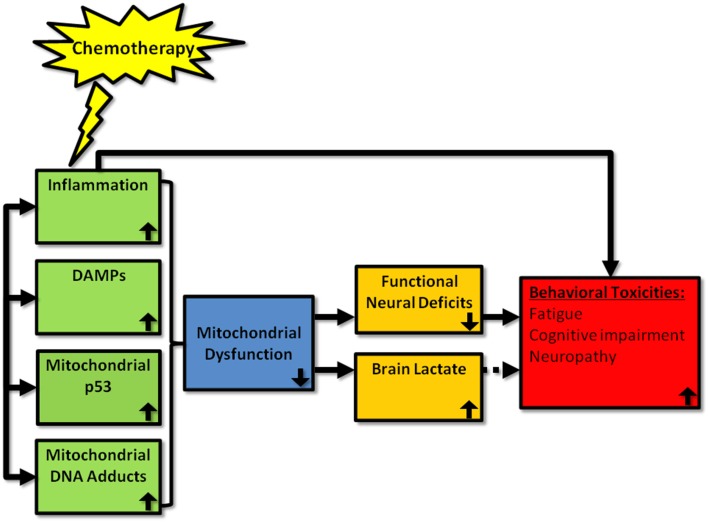
**Proposed mechanisms by which chemotherapy can induce behavioral toxicities**. Chemotherapy has been shown to induce peripheral inflammation, DAMP, mitochondrial p53, and mitochondrial adducts. We propose that chemotherapy also induces these processes within the brain, which leads to mitochondrial dysfunction. This, in turn, leads to neural deficits and increased brain lactate. Depending upon the localization of these neuronal deficits in the brain, behavioral toxicities—such as fatigue, cognitive impairment, and neuropathy—are likely to emerge. Whether lactate production is a byproduct or inducer of symptoms is as yet unclear. Further, it is possible that chemotherapy-induced inflammation may also act to induce behavioral toxicities via non-mitochondrial related pathways. Up and down arrows represent the direction of the effect

Promising alternative mechanisms for chemotherapy-induced behavioral toxicities are DAMPs and the bioenergetics status of cells of the CNS (Figure 1). These avenues of investigation are growing rapidly and need to be integrated into the field more widely. In regards to DAMPs, most work has been conducted in relation to HMGB1 but a range of other DAMPs are known to be activated in response to chemotherapies, and the activation of specific DAMPs may be chemotherapy agent-specific. The prospect that DAMPs may be a major player in chemotherapy-induced behavioral symptoms is particularly convincing given that they often cause downstream production of pro-inflammatory cytokines which may suggest that the focus of many of us in the field on neuroinflammation *per se* has been a matter of “putting the cart before the horse.” The same may also be said for the field of bioenergetics and symptoms of chemotherapy given the relationship between mitochondrial dysfunction and inflammation. However, the evidence that is emerging also indicates that alterations in mitochondrial energy metabolism and production of metabolites such as lactate are likely to contribute to cancer-related symptoms in an independent fashion also. Clearly, the literature is currently somewhat scarce for DAMPs and mitochondrial dysfunction in the field of chemotherapy-induced behavioral toxicities but they represent exciting new avenues of research that should complement our understanding of the mechanisms at the origin of cancer-related symptoms.

### Conflict of interest statement

The authors declare that the research was conducted in the absence of any commercial or financial relationships that could be construed as a potential conflict of interest.
